# Hormonal Relationships to Bone Mass in Elderly Spanish Men as Influenced by Dietary Calcium and Vitamin D

**DOI:** 10.3390/nu5124924

**Published:** 2013-12-04

**Authors:** Jose M. Moran, Luis Gonzalez Lopez-Arza, Jesus M. Lavado-Garcia, Maria Pedrera-Canal, Purificacion Rey-Sanchez, Francisco J. Rodriguez-Velasco, Pilar Fernandez, Juan D. Pedrera-Zamorano

**Affiliations:** Metabolic Bone Diseases Research Group, School of Nursing and Occupational Therapy, University of Extremadura, Caceres 10003, Spain; E-Mails: jmmorang@unex.es (J.M.M.); mvglez@unex.es (L.G.L.-A.); jmlavado@unex.es (J.M.L.-G.); mariapedreracanal@gmail.com (M.P.-C.); prey@unex.es (P.R.-S.); fcorodriguezv@unex.es (F.J.R.-V.); piferf@enfermermundi.com (P.F.)

**Keywords:** PTH, vitamin D, peripheral bone mineral density, QUS

## Abstract

We aim to evaluate whether calcium and vitamin D intake is associated with 25-hydroxyvitamin D (25-OH-Vitamin D_3_) and parathyroid hormone (PTH) serum concentrations or is associated with either the phalangeal dual energy X-ray absorptiometry (pDXA) or the quantitative bone ultrasound (QUS) in independent elderly men. Serum PTH and 25-OH-Vitamin D_3_ were measured in 195 healthy elderly men (mean age: 73.31 ± 5.10 year). Food intake was quantified using a dietetic scale. Participants with 25-OH-Vitamin D_3_ levels ≥ 30 ng/mL (75 nmol/L) and a calcium intake of 800–1200 mg/day exhibited the lowest PTH levels (41.49 ± 16.72 ng/mL). The highest PTH levels (75.60 ± 14.16 ng/mL) were observed in the <30 ng/mL group 25-OH-Vitamin D_3_ with a calcium intake >1200 mg/day. No significant differences in the serum PTH levels based on the serum 25-OH-Vitamin D3 levels were observed among participants with a calcium intake of 800–1200 mg/day. Serum PTH was inversely correlated with serum 25-OH-Vitamin D_3_ in the entire patient sample (*r* = −0.288, *p =* 0.019). No differences in any of the three densitometry techniques were observed between any of the age groups in the 800–1200 mg/day and >1200 mg/day calcium intake groups. PTH levels correlate negatively with serum 25-OH-Vitamin D_3_ levels, and neither calcium nor vitamin D intake exert a strong influence on either of the two parameters.

## 1. Introduction

Calcium and vitamin D are two of the most important nutrients for bone health, and both nutrients play a pivotal role in the prevention of osteoporotic fractures [1]. For elderly men (>65 year), the recommended dietary allowances (RDAs) for calcium and vitamin D are 800 mg/day and 15 µg/day, respectively [2]. Spain is situated in a geographical area with abundant sun exposure; however, the vitamin D status in the elderly might require supplementation because the elderly population in Spain consumes less than the RDA for both calcium and vitamin D [3,4,5]. Additionally, calcium absorption may be less efficient in the elderly [6]. In Europe, serum 25-OH-Vitamin D_3_ is measured as a reflection of the total vitamin D derived from food and dietary supplements and synthesized in the skin. Serum 25-OH-Vitamin D_3_ values below 50 nmol/L (20 ng/mL) have been defined as deficient [7]. Vitamin D deficiency causes malabsorption of calcium and osteomalacia and increases the risk of fractures [8]. Additionally, factors other than calcium and vitamin D can influence calcium homeostasis. Vitamin D deficiency is often associated with secondary hyperparathyroidism, which contributes to age-related bone loss [9] and other non-skeletal conditions [10]. In general, it is accepted that up to a certain level of 25-OH-Vitamin D_3_, there is a negative relationship between 25-OH-Vitamin D_3_ and PTH, and beyond this point, little further decrease in PTH is observed [10]. This putative threshold level of serum 25-OH-Vitamin D_3_ (~78 nmol/L; 31 ng/mL) [11] could be considered an inflection point for correct calcium homeostasis and would indicate the range of vitamin D sufficiency [12,13]. However, other authors have reported different inflection points that vary from ~50 nmol/L (20 ng/mL) [14] to ~110 nmol/L (44 ng/mL) [15], and although key reviews in the field have suggested a consensus threshold of 75 nmol/L (30 ng/mL) [12,16], this has been questioned and a more accurate threshold of 20 ng/mL (50 nmol/L) of serum 25-OH-Vitamin D_3_ has recently been adopted [17] to define vitamin D insufficiency as it relates to bone. 

The relationship between calcium intake and the serum 25-OH-Vitamin D_3_ threshold has been studied, and low calcium intake has been proposed to be irrelevant to maintaining normal PTH levels in subjects with 25-OH-Vitamin D_3_ levels exceeding 25 nmol/L (10 ng/mL). However, high calcium intake (>1200 mg/day) is not sufficient to maintain ideal serum PTH when the vitamin D status is insufficient [13]. The relationship between 25-OH-Vitamin D_3_ and PTH may also be affected by age [18,19] and disease [20].

Large cross-sectional studies have examined the importance of calcium intake and serum 25-OH-Vitamin D_3_ on bone mineral density (BMD) by central DXA [21]. In a study of men in the U.S. with a mean calcium intake of 800 mg/day, the authors observed no association with femoral BMD at any concentration of 25-OH-Vitamin D_3_ [21]. The impact of calcium intake and both serum 25-OH-Vitamin D3 and PTH concentrations on peripheral BMD and quantitative ultrasound (QUS) has not yet been addressed.

We hypothesize that calcium and vitamin D intake is associated with 25-OH-Vitamin D_3_ and PTH serum concentrations and either pBMD or QUS in independent elderly men from a rural area of southwestern Spain.

## 2. Experimental Section

The study was performed between January and December 2011. The mean amount of total sunlight exposure was 235.83 h. A total of 195 healthy elderly men were selected for the study. The participants had no dietary restrictions, neurological impairments, or physical handicaps. None of the subjects were taking medications that could interfere with calcium metabolism. All of the participants were from the Health District of Llerena-Zafra, (Extremadura) Spain. All of the participants provided written informed consent, and the Office for the Protection of Research Risks of the University of Extremadura approved the research in accordance with the Helsinki Declaration of 1975.

Height measurements were made using a Harpender stadiometer (Harpender Pfifter 450, Carlstandt, NJ, USA), and weights were measured on a biomedical balance.

### 2.1. Nutrient Intake

Nutrient intake was quantified using a dietetic scale, measuring cups, cans, small bottles, and spoons on the basis of current 7-day dietary records, as in previous studies [22].

### 2.2. Ultrasound Studies

Bone status was assessed using an ultrasound device, model DBM Sonic 1200 (IGEA, Carpi, Italy), which measures amplitude-dependent speed of sound (Ad-SOS) in meters per second at the phalanges, as previously described [22]. Instrument precision was determined from three measurements in eight subjects at time intervals not exceeding 21 days. The coefficient of variation (CV) was 0.77%. The inter-observer CV was 1.1%.

The Cuba Clinical (McCue Ultrasonics, UK) dry ultrasound portable device was used to measure broadband ultrasound attenuation (BUA), expressed in dB/MHz. The measurements were performed at both heels. For each individual, the mean values of BUA were calculated. Measurement precision, based on regular weekly phantom measurements, was expressed as a CV of 2.97% for BUA. The interobserver coefficient of variation was <1%.

### 2.3. Peripheral Bone Mineral Density Study

pBMD of the phalanges was calculated using the middle finger of the non-dominant hand and was measured using AccuDXA (Schick technologists, Long Island City, NY, USA). Two trained technicians performed all scans, and calibration of the machine was performed on each scanning day to ensure the accuracy of BMD measurements. The precision error CV of the BMD estimations was 0.98%. The interobserver coefficient of variation was <1%.

### 2.4. Analytical Studies

No coffee, tea, smoking, alcohol, or exercise were permitted 24 h before the investigation. The hematological and biochemical studies were performed on blood samples taken during a fasting state at 8:00 a.m.

The blood samples were centrifuged, and the serum was stored at −20 °C until analysis. All of the samples were analyzed in the same assay to eliminate inter-assay variation. Assay reproducibility was determined by assaying four samples five times in five different runs. The CV between runs was determined by components of variance [23]. In every case, the CV was less than 6%. PTH was measured using the Intact PTH IRMA kit (Nichols Institute Diagnostic, San Juan Capistrano, CA, USA); the intra-assay and inter-assay CV were each <5%. The serum 25-OH-Vitamin D_3_ levels were measured with the 25OH-vit. D_3_-RIA-CT kit of Biosource Europe, S.A (Zoning Industriel, Nivelles, Belgium); the intra-assay and inter-assay CV were each <8%.

### 2.5. Statistical Studies

All of the values are expressed as the mean ± SD or the mean with the 95% CI. The normal distribution of the data was confirmed by calculating skewedness and kurtosis before applying standard tests. The groups (when appropriate) were compared using analysis of variance to determine the differences. A minimum *p*-value of <0.05 was the necessary condition for statistical significance. Regression and correlation analysis were used, when appropriate, to examine the relationships between continuous variables. Stepwise multiple linear regression analysis was executed to estimate the linear relationship between dependent variables (serum PTH, serum 25-OH-Vitamin D_3_, pBMD, QUS) and various independent variables. These studies were performed using SPSS 19.0.

## 3. Results

**Figure 1:**
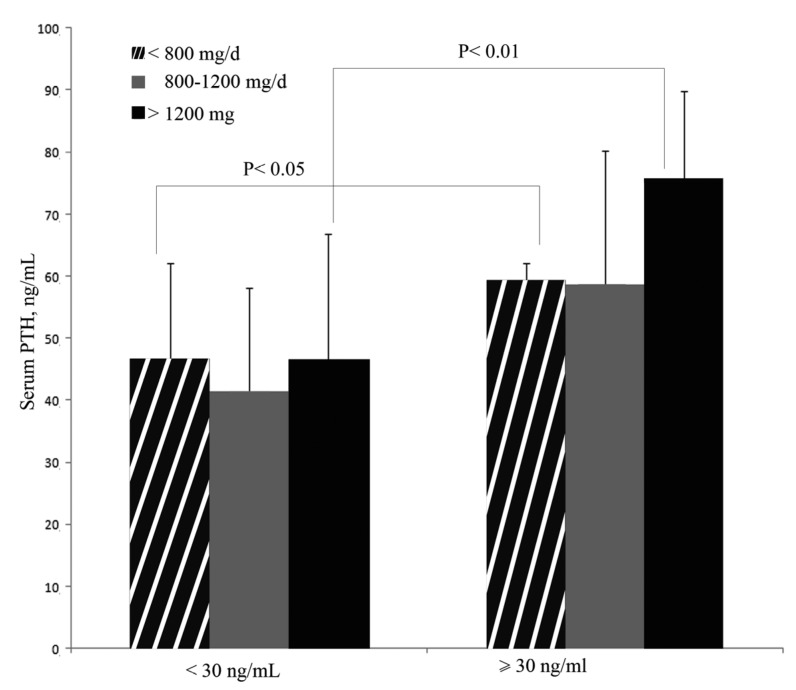
The mean serum PTH levels according to serum 25-OH-Vitamin D_3_ and calcium intake. The data are presented as the means (SD). To convert ng/mL to nmol/L, multiply by 2.496.

The baseline biochemical characteristics and nutrient intake of the 195 men are presented in Table 1. The mean (SD) values for vitamin D and calcium intake and serum PTH by age groups are presented in Table 2. Calcium intake in the 65–69 year group (663.59 mg/day ± 112.00) was significantly higher than that observed in the 70–74 year group (488.30 mg/day ± 125.00) (*p =* 0.048). No further significant differences were observed among groups for any of the studied variables (*p* > 0.05 in all cases). Figure 1 presents serum PTH levels according to serum 25-OH-Vitamin D_3_ levels (<30 ng/mL and ≥30 ng/mL) and calcium intake. The lowest serum PTH levels (41.39 ng/mL ± 16.72) were observed in the group with a serum 25-OH-Vitamin D_3_ >30 ng/mL and a calcium intake of 800–1200 mg/day. The highest PTH levels (75.60 ng/mL ± 14.16) were observed in the group with serum 25-OH-Vitamin D_3_ <30 ng/mL and calcium intake >1200 mg/day. Significant differences were observed between the groups with calcium intakes <800 mg/day and >1200 mg/day based on the serum 25-OH-Vitamin D_3_ levels. Serum PTH levels were in both cases higher in the group with serum 25-OH-Vitamin D_3_ <30 ng/mL (*p* < 0.05 for a calcium intake <800 mg/day and *p* < 0.01 for a calcium intake > 1200 mg/day, respectively). No significant differences in the serum PTH levels were observed between participants with a calcium intake of 800–1200 mg/day based on the serum 25-OH-Vitamin D_3_ levels (<30 ng/mL and ≥30 ng/mL).

**Table 1:** Anthropometric, biochemical and nutrient intake data in the studied simple.

	Mean (SD)	Range
Age (y)	73.31 (5.10)	65.00–88.00
Weigth (kg)	80.69 (11.67)	52.00–124.00
Heigth (m)	1,65 (0.06)	1.45–1.79
BMI (kg/m^2^)	29,73 (4.03)	20.76–46.10
PTH (ng/mL)	49.69 (25.18)	2.50–189.10
Serum Vitamin D (ng/mL)	43.03 (17.98)	5.00–119.00
Vitamin D intake (µg/day)	3.90 (4.78)	0.00–437
Calcium intake (mg/day)	535.81 (358.11)	112.00–2457.00

SI conversion: To convert serum 25-OH-Vitamin D3 to nmol/L, multiply by 2.496.

**Table 2:** Calcium and Vitamin D intake and serum 25-OH-Vitamin D_3_ and PTH by age group.

	Age group							
	65–69 (*n* = 45)		70–74 (*n* = 81)		75–80 (*n* = 52)		>80 (*n* = 21)	
	Mean (SD)	Range	Mean (SD)	Range	Mean (SD)	Range	Mean	Min
Calcium intake (mg/day)	663.58 (466.13)	112.00–2457.00	488.30 (305.53)	125.00–1367.00	501.22 (318.55)	120.00–1452.00	527.00 (327.84)	146.00–1283.00
Vitamin D intake (µg/day)	5.47 (7.73)	0.02–43.7	3.54 (3.50)	0.16–16.5	3.45 (3.16)	0.11–30.4	3.04 (3.37)	0.00–10.4
Serum 25-OH-Vitamin D3 (ng/mL)	43.13 (16.63)	11.00–82.00	44.21 (17.69)	8.00–119.00	42.98 (21.07)	5.00–105.00	38.61 (13.45)	20.00–65.00
Serum PTH (ng/mL)	50.73 (26.64)	17.70–164.70	46.61(22.73)	14.20–52.11	52.11 (28.34)	2.50–189.10	52.70 (22.70)	20.00–97.90

SI conversions: To convert serum 25-OH-Vitamin D3 to nmol/L, multiply by 2.496; Calcium intake 65–69 *vs*. 70–74 *p =* 0.048, calculated by ANOVA with Bonferroni adjustment.

We analyzed our data to compare the effects of dietary calcium and vitamin D on serum PTH. The serum PTH concentration did not correlate with calcium intake in the total sample population (*p =* 0.694).

When the population was divided into two groups by serum 25-OH-Vitamin D_3_ levels (<30 ng/mL and ≥30 ng/mL), the lack of correlation persisted in both groups (≥30 ng/mL, *p =* 0.845; and <30 ng/mL, 0.817).

The serum 25-OH-Vitamin D_3_ levels also failed to correlate with calcium intake (*p =* 0.238) in the total sample population. When the population was divided into two groups by serum 25-OH-Vitamin D_3_ levels (<30 ng/mL and ≥30 ng/mL), the absence of correlation persisted in both groups (≥30 ng/mL, *p =* 0.097; and <30 ng/mL, 0.087). We also failed to identify any association between vitamin D intake and serum 25-OH-Vitamin D_3_ in the sample population (*p =* 0.547).

**Figure 2:**
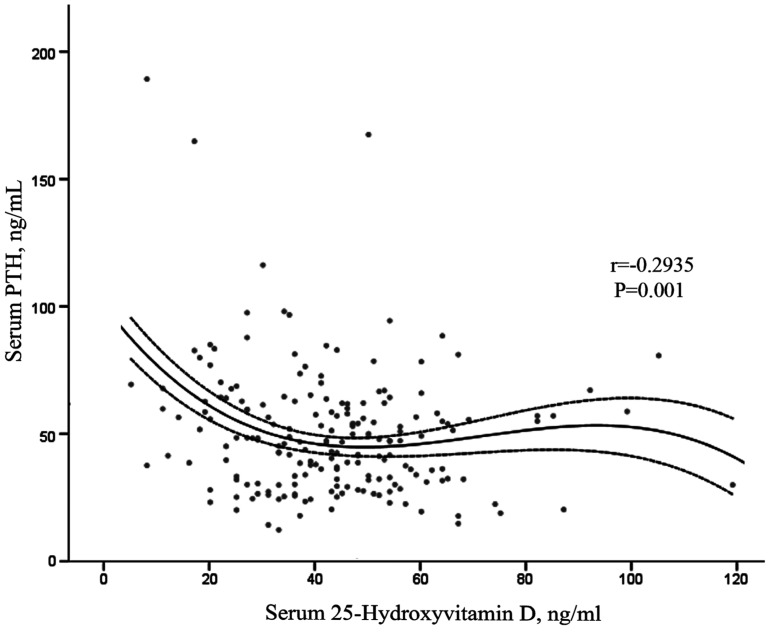
Correlation between serum PTH and serum 25-OH-Vitamin D_3_ in 195 men. Serum PTH was inversely correlated with serum 25-OH-Vitamin D_3_. The solid line is the cubic reqikgression of the data (*r*^2^ = 0.086). Dotted lines represent IC 95% for the mean.

Serum PTH was inversely correlated with serum 25-OH-Vitamin D_3_ in the total sample population (Figure 2). This relationship persisted after adjusting for confounding factors (age and vitamin D intake) (*r* = −0.170, *p =* 0.019). When the subjects were divided by serum 25-OH-Vitamin D_3_ levels (<30 ng/mL and ≥30 ng/mL), the correlation between the serum PTH and serum 25-OH-Vitamin D_3_ remained negative and increased in slope for subjects with a serum 25-OH-Vitamin D_3_ <30 ng/mL (*r* = −0.288, *p =* 0.049), whereas the correlation disappeared for subjects with a serum 25-OH-Vitamin D_3_ ≥30 ng/mL (*p =* 0.651).

**Figure 3:**
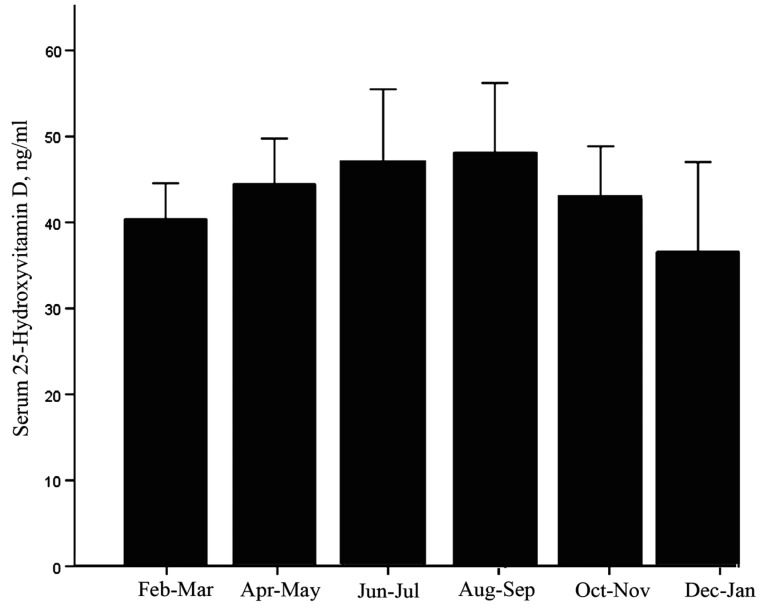
Seasonal variation in serum 25-OH-Vitamin D_3_ in the study sample. The data are presented as the means (CI 95%). No significant differences were identified by ANOVA (*p =* 0.260).

We did not observe a significant effect of the season on the serum 25-OH-Vitamin D_3_ levels as shown by ANOVA (*p =* 0.260). There were no differences when the participants were divided by serum 25-OH-Vitamin D_3_ levels (<30 ng/mL and ≥30 ng/mL; *p =* 0.658 and *p =* 0.156, respectively). Figure 3 presents the mean serum 25-OH-Vitamin D_3_ levels at two-month intervals throughout the year. 

Vitamin D deficiency, defined as a serum 25-OH-Vitamin D_3_ concentration below 20 ng/mL (50 nmol/L), was observed in 9.74% (*n* = 19) of the sample population (IC 95%, 6.32%–14.71%). Vitamin D insufficiency, serum 25-OH-Vitamin D_3_ between 21 and 29 ng/mL (52.5–72.5 nmol/L) was observed in 13.85% (*n* = 27) (IC 95%, 9.70%–19.40%) of the sample population. Secondary hyperparathyroidism (serum PTH ≥ 65 pg/mL) was observed in 13.79% of the subjects (IC 95%, 13.79%–24.74%).

We next considered the putative relationship among serum PTH, serum 25-OH-Vitamin D_3_ and calcium and vitamin D intake and the pBMD and QUS of the phalanges or calcaneus. We only observed a significant relationship between vitamin D intake and pBMD (*r* = 0.244, *p =* 0.001). None of the remaining relationships were significant. The correlation between pBMD and vitamin D intake disappeared after controlling for confounding factors (age and weight) (*p =* 0.474). When the subjects were divided by serum 25-OH-Vitamin D_3_ levels (<30 ng/mL and ≥30 ng/mL), calcium intake correlated positively with QUS at the phalanges (*r* = 0.177, *p =* 0.042) in the ≥30 ng/mL serum 25-OH-Vitamin D_3_ group, and vitamin D intake correlated with pBMD (*r* = 0.300, *p* < 0.0001). No significant relationships were observed in the ≥30 ng/mL serum 25-OH-Vitamin D_3_ group. The detected correlation between pBMD and vitamin D intake disappeared after controlling for confounding factors (age and weight).

**Figure 4:**
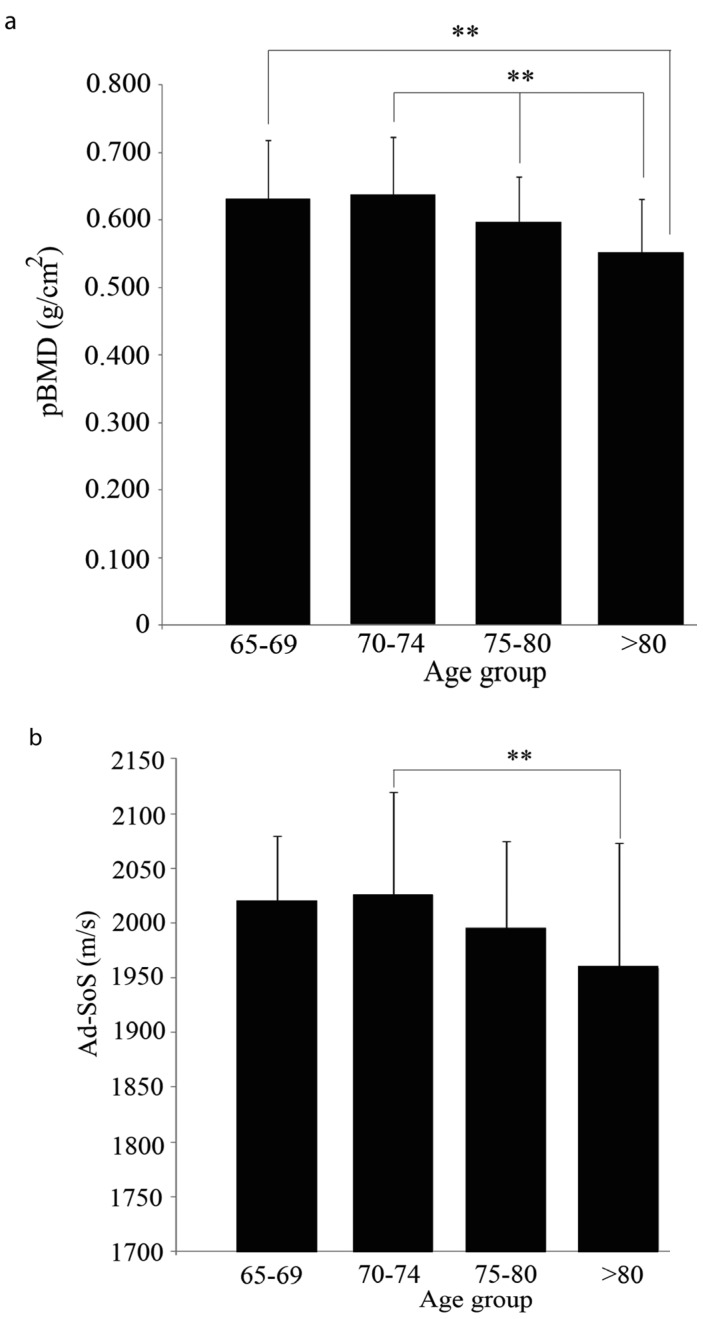
Bone mass in the <800 mg/day calcium intake group by age group. (**a**) pBMD and (**b**) QUS at the phalanges. ** (*p* < 0.01).

The effect of calcium intake on the pBMD and the QUS was also studied. In the >74 year age group with a calcium intake <800 mg/day, the pBMD decreased significantly with age (Figure 4a). The QUS at the phalanges in the 70–74 year group was higher than that observed in the >80 year age group (*p* < 0.01) (Figure 4b). No differences were observed between any age groups in the 800–1200 mg/day and >1200 mg/day calcium intake group with any of the three techniques. 

The main determinants of serum PTH and serum 25-OH-Vitamin D_3_ were examined by multiple regression analysis. For serum PTH and serum 25-OH-Vitamin D_3_, the variables entered in the model as independent predictors included age, weight, BMI, serum 25-OH-Vitamin D_3_, calcium intake and vitamin D intake. Serum 25-OH-Vitamin D_3_ represented the sole variable that contributed significantly to PTH variance (β = −0.239, *F* = 4.231, *p =* 0.041). Similarly, serum PTH was the sole variable that explained serum 25-OH-Vitamin D_3_ variance (β = −1.113, *F* = 4.231, *p =* 0.041). 

We also analyzed the main determinants of pBMD and both phalangeal and calcaneal QUS. None of the variables were found to be determinants of QUS at the calcaneus in the studied men. QUS at the phalanges was determined by both age (β = −5.024, *F* = 14.809, *p* < 0.0001) and BMI (β = −5.637, *F* = 14.809, *p =* 0.001). Finally, pBMD variance was explained by age (β = −0.004, *F* = 13.878, *p* < 0.0001) and vitamin D intake (β = 3.554, *F* = 13.878, *p =* 0.003).

## 4. Discussion

A considerable number of elderly Europeans fail to meet the lowest European RDAs for some nutrients and might be at risk of vitamin and mineral deficiencies [24]. In our sample, the average mean calcium intake of 535.81 ± 358.11 mg/day was lower than the RDA and also the dietary vitamin D intake (3.90 ± 4.78 µg/day) [2]. These results are similar to those found by previous studies which reported vitamin D intakes lower than the RDA in the elderly in Spain [3,25,26].

Accordingly to the recommendations of The Endocrine Society in 2011 [27] we observed a vitamin D deficiency in 9.74% of our study subjects (IC 95%, 6.32%–14.71%). This value is lower than the observed in Europe [11,28,29] and outside Europe [30], likely indicating a significant role of skin pigmentation, sunshine and dietetic patterns in Spain and other Mediterranean countries [29]. Studies in different geographical regions worldwide have described seasonal variations in serum 25-OH-Vitamin D_3_, with the lowest levels measured during the winter [31]. However, we did not observe differences in our area. Extremadura has the highest rate of sunshine in Spain during the months of July and August, receiving 2830 sunshine h/year (1978 to 2000 (AEMET, Spanish Meteorological Office)). Thus, it is possible that Extremadura’s elderly men are able to synthesize sufficient vitamin D in their skin to cover their daily needs. Our results are higher than other recent studies that describe the prevalence of vitamin D deficiency in elderly men in Spain to be 4.5% [32] or 5.3% [33]. However, these studies did not follow the thresholds recommended by The Endocrine Society, and in both studies vitamin D deficiency was established with a serum 25-OH-Vitamin D_3_ level below 10 ng/mL (25 nmol/L) and therefore lower figures could be expected. 

Secondary hyperparathyroidism was observed in 13.79% (IC 95%, 13.79%–24.74%) of the sample, whereas vitamin D insufficiency/deficiency was observed in 32.65% (IC 95% 26.5%–40.59%) of the sample. It has been previously reported that of elderly patients who suffered hip fractures and had a serum 25-OH-Vitamin D_3_ < 12 ng/mL, only half the patients had evidence of secondary hyperparathyroidism, whereas the rest exhibited low to normal levels of PTH or functional hypoparathyroidism [34]. Other micronutrients, like magnesium, might play a role in this relationship. It has been proposed that magnesium deficiency is associated with impaired PTH secretion [35]. We did not address magnesium levels in our sample. However, the magnesium intake in the sample (170.53 ± 74.99 mg/day) was below the recommended RDA for Spanish elderly men (350 mg/day) [2].

Serum PTH is one of the main determinants of bone remodeling [10], and its association with the intake of calcium and vitamin D, needs to be addressed. We observed a strong inverse relationship between serum PTH concentrations and 25-OH-Vitamin D_3_. Different thresholds levels of serum 25-OH-Vitamin D_3_ have been proposed to indicate serum 25-OH-Vitamin D_3_ insufficiency [10,14,15], and it has even been proposed that this value may not exist [36]. We observed that the threshold of 30 ng/mL (75 nmol/L) serum 25-OH-Vitamin D_3_ clearly divides our sample in two groups. In the group with a serum 25-OH-Vitamin D_3_ concentration below 30 ng/mL, the PTH concentrations and 25-OH-Vitamin D_3_ were inversely correlated, whereas concentrations of serum 25-OH-Vitamin D_3_ greater than 30 ng/mL did not affect serum PTH concentrations in elderly Spanish men. 

Studies in postmenopausal women identified a strong influence of age and calcium intake [37] in serum PTH. Steingrimsdottir *et al*. in a cross-sectional study with men from Iceland, also observed a correlation with calcium intake [13]. However, in their sample, vitamin D intake and calcium intake were over the recommended levels in all of the age groups, whereas in our sample, a clear deficiency was observed in the intake of both nutrients. This discrepancy may partly explain our findings, as the effect of calcium intake may be less apparent when it is under the recommended ranges. Nevertheless, increasing calcium intake appears to cause increased suppression of PTH only in subjects with low vitamin D status [13,38], an effect that we have observed in Spanish elderly men, as calcium intake >1200 mg/day is somehow related to the highest level of serum PTH detected in the group with a serum 25-OH-Vitamin D_3_ <30 ng/mL. Our results indicate significant differences in PTH when calcium intake is misbalanced (<800 mg/day or >1200 mg/day) based on the serum 25-OH-Vitamin D_3_ levels. Because PTH levels in healthy subjects with an inadequate level of serum 25-OH-Vitamin D3 increases with a calcium intake >1200 mg/day or <800 mg/day, we suggest the importance of calcium intake between the recommended ranges for Spanish elderly men. Further investigations are necessary to correctly evaluate this effect in such a population. The association among 25-OH-Vitamin D_3_, PTH and central BMD has been addressed previously. In general, studies indicate that lower 25-OH-Vitamin D_3_ levels correlate with higher PTH levels and lower BMD values at the hip [13,21]. Recently, it has been proposed that calcium intake correlates positively with BMD at the femoral neck, even at low 25-OH-Vitamin D_3_ concentrations [39]. A positive relationship has also been described in males (50 ± 9.6 year (range 26–76)) between 25-OH-Vitamin D_3_ and BMD at the spine and calcium intake but not with serum PTH and vitamin D intake [40]. However, Marwaha *et al.* failed to detect any correlation between 25-OH-Vitamin D_3_ and either central or peripheral BMD in a sample of 792 Indian males aged 58.0 ± 10.3 year [41]. In the present study, multiple regression analysis revealed a significant correlation between vitamin D intake and pBMD in men. Other authors also failed to find a significant correlation between vitamin D status and BMD in men but not in women [42,43]. Taken together, these studies suggest a weak association of 25-OH-Vitamin D_3_ with BMD in men that becomes particularly strong in postmenopausal women.

Vitamin D- and calcium-supplemented elderly care residents who exhibit increased 25-OH-Vitamin D_3_ levels in the adequate range and significantly reduced PTH levels do not exhibit any relationship between serum 25-OH-Vitamin D_3_ or PTH and bone mass as measured by QUS at the heel [44]. Similarly, we have not observed any correlation between QUS at the heel and any of the studied variables.

Both peripheral BMD at the phalanges and QUS at the phalanges exhibited significant decreases associated with age in the <800 mg/day calcium intake group, but we failed to detect any association of this effect with the serum level of PTH or 25-OH-Vitamin D_3_. 

In the present pilot study, we recognize several limitations. First, our study comprised a small sample size, with statistical power limitations in a few of the reported results, and nevertheless, we cannot generalize our findings to the broader community based on this pilot study alone. Second, correlations demonstrated in our study should be cautiously interpreted since notwithstanding the fact that, although significant, they are on the order of a small to moderate association. The results of the present pilot study warrant additional research involving larger numbers of subjects and longitudinal assessments. It would be also of considerable interest to re-evaluate the presented data from the central BMD point of view in Spanish elderly men.

## 5. Conclusions

In conclusion, in Spanish elderly men, serum PTH levels correlate negatively with serum 25-OH-Vitamin D_3_ without any strong influence of calcium or vitamin D intake on the two parameters. Further investigations are needed to evaluate the effect of adequate calcium intake (between 800–1200 mg/day) on PTH control in such a population when the serum 25-OH-Vitamin D_3_ is suboptimal. Therefore, vitamin D supplementation in the Extremadura elderly population may occasionally be recommended as a contribution to reducing the risk of alterations in bone health.
